# Promotion of microRNA-146a by histone deacetylase 4 silencing contributes to radiosensitization of esophageal carcinoma

**DOI:** 10.1186/s12967-021-03171-z

**Published:** 2022-02-22

**Authors:** Zhonghua Lu, Yifei Yun, Yutong Zhang, Yao Ou, Meihua Wang

**Affiliations:** 1 Department of Radiotherapy, Changzhou Tumor Hospital, Soochow University, Changzhou, 213000 China; 2 Department of Pathology, Changzhou Tumor Hospital, Soochow University, No. 68, Honghe Road, Xinbei District, Changzhou, 213000 Jiangsu China

**Keywords:** Esophageal carcinoma, Histone deacetylase 4, miR-146a, IRAK1, Radioresistance, In vitro and in vivo studies

## Abstract

**Background:**

Histone deacetylases (HDACs) have been identified to be implicated in the carcinogenesis and cancer progression. The present study was performed to probe into the effect of HDAC4 on radioresistance of esophageal carcinoma (EC).

**Methods:**

The expression of HDAC4 in responders and non-responders to radiotherapy was characterized by RT-qPCR, immunohistochemistry, and Western blot analysis. EC cells were exposed to continuous fractionated X-ray irradiation, and their proliferation and apoptosis were evaluated by means of colony formation assay and flow cytometry based Annexin V-FITC/PI apoptosis assay in response to HDAC4 overexpression or silencing. Mechanistic investigation was conducted by means of in silico analysis and dual-luciferase reporter gene assay. Tumor xenografts derived from radioresistant EC cells were exposed to local X-ray irradiation in vivo for validation.

**Results:**

High expression of HDAC4 was detected in either tumor tissues derived from radiotherapy responders or radioresistant EC cells. Loss of HDAC4 contributed to suppressed proliferation and enhanced apoptosis of radioresistant EC cells. Moreover, our findings revealed that HDAC4 conferred radioresistance of EC by downregulating microRNA-146a (miR-146a). Interleukin-1 receptor-associated kinase 1 (IRAK1) was a target of miR-146a, and its knockdown promoted radiosensitivity. Silencing of HDAC4 radiosensitized EC cells both in vitro and in vivo via the miR-146a/IRAK1 axis.

**Conclusion:**

Hence, loss of HDAC4 upregulated miR-146a to limit radioresistance. This study aids in the better understanding about mechanism responsible for radioresistance of EC.

**Supplementary Information:**

The online version contains supplementary material available at 10.1186/s12967-021-03171-z.

## Background

Esophageal cancer (EC) represents the eighth most commonly occurring cancer, as well as the sixth most frequent cause of cancer related death worldwide [1]. Esophageal cancer can be divided into two main subtypes: esophageal adenocarcinoma and esophageal squamous cell carcinoma (ESCC) [2], between which ESCC accounts for about 90% of EC cases [3]. Although radiotherapy is considered as an effective and well-established treatment for ESCC, patients still suffer relapse and treatment failure due to acquired radioresistance of ESCC cells [4]. Thus, there is an urgent need to investigate the molecular mechanisms underlying radioresistance of ESCC cells, aiming to improve the effectiveness of radiation treatment.

Recently, histone deacetylases (HDACs) have emerged as a factor involved in the carcinogenesis and progression of multiple human cancers, by virtue of its effects on gene transcription [5]. The effects of the HDAC family member HDAC4 on radioresistance of cancer cells have been explored [6]. Indeed, HDAC4 is essential for the progression of EC, thus presenting as a therapeutic target for EC [7]. Wang et al*.* have revealed that HDAC regulates the expression of the microRNA-146a (miR-146a) [8], which is also implicated in the occurrence and development of ESCC [9]. miRNAs are a class of small noncoding RNAs, which have significant impacts on various biological processes, including cellular proliferative and apoptotic potential, via post-transcriptional regulation of gene expression [10]. Moreover, miRNAs exert effects on cancer radioresistance by simultaneously regulating several oncogenic pathways that may affect radiation response [11]. While there is hitherto little data on miR-146a expression in radioresistance of ESCC cells, miR-146a can target the interleukin-1 receptor-associated kinase 1 (IRAK1) and negatively regulate its expression [12]. IRAK1 is known to be upregulated in human cancers in relation to tumor progression [13]. A recent study has revealed effects of miR-146a and IRAK1 in the development of cervical cancer [14]. However, their specific mechanisms in EC cells resistant to radiotherapy remain largely unknown. Based on the above findings, we hypothesized that HDACs regulated the expression of miR-146a and its target IRAK1 to affect radioresistance of EC cells. We carried out a series of studies to test the mechanisms by which HDAC4 affects EC radioresistance through regulation of miR-146a-targeted IRAK1.

## Materials and methods

### Ethics statement

Written informed consents were obtained from all patients prior to the study. The protocols of this study were approved by the Ethic Committee of Changzhou Tumor Hospital, Soochow University. The protocols of animal experiments were approved by the Institutional Animal Care and Use Committee of Changzhou Tumor Hospital, Soochow University.

### Tissue collection

The ESCC tissues were obtained from 82 patients who had undergone surgery and postoperative radiotherapy in Changzhou Tumor Hospital, Soochow University from June 2015 to June 2016. When induction conventional radiotherapy was completed, patients underwent endoscopic ultrasonography and biopsy. According to World Health Organization criteria for evaluating the progression of solid tumors [15], the patients were divided into the Complete Remission (CR), Partial Remission (PR), Stable Disease (SD), and Progressive Disease (PD) groups, where the composite of CR and PR (n = 46) were considered to be responders and the composite of resistant SD and PD cases (n = 36) were designated as resistant. A follow-up survey of 82 patients was conducted for three years.

### Plasmid construction and cell treatment

EC cell lines KYSE30 (bio-106132, Biobw Biotechnology Co., Ltd., Beijing, China), KYSE140 (CL0437, Hunan Fenghui Biotechnology Co., Ltd., Changsha, China), KYSE150 (bio-107428, Biobw Biotechnology Co., Ltd.), and KYSE180 (C7130, Shanghai GuanDao Biological Engineering Co., Ltd., Shanghai, China) and a human normal esophageal epithelial cell line NE1 (SHC926, Saihongrui Biotechnology Co., Ltd., Nanjing, China) were used in this study. The EC cells were incubated in Roswell Park Memorial Institute 1640 medium (Gibco, Carlsbad, CA) supplemented with 10% fetal bovine serum and 100 U/mL streptomycin. Subsequently, cells were cultured in an incubator (Thermo Fisher Scientific, Rockford, IL) with 5% CO_2_ at 37 °C.

NE1 cells were incubated in the mixed medium of EpiLife medium containing growth supplement (Invitrogen) and 60 µM Ca^2+^, and serum-free medium at a ratio of 1: 1. Cells were inoculated into 6-well plates at a density of 1 × 10^5^ cells/well and incubated for 24 h. Upon reaching 60% confluence, cells were transduced following instructions of Lipofectamine 2000 (Invitrogen). short hairpin RNAs (shRNAs) were synthetized by Shanghai Sangon Biotechnology Co., Ltd. (Shanghai, China). Cells were treated with scramble shRNA, shRNA against HDAC4 (sh-HDAC4), sh-IRAK1, empty vector (pcDNA3.1), and overexpressed HDAC4 (oe-HDAC4) (pcDNA-HDAC4). The miR-146a mimic and inhibitor and plasmids harboring shRNA or pcDNA3.1 vector were synthetized by Shanghai GenePharma Co., Ltd. (Shanghai, China).

### Construction of radioresistant EC cells

EC KYSE30 cells were irradiated by X-rays from as linear accelerator under the following conditions: X-ray tube voltage 150 kV, X-ray tube current 20 mA, air kerma rate at the probe position 7.180 Gy/m, air kerma rate at irradiated object 4.81 Gy/m, distance between focus and samples 350 mm, and dose at first irradiation of 1 Gy. After each irradiation, cells were cultured in the fresh culture medium and put back into the incubator. When the cells grew to 90% confluence, they were passaged and paved the plates. Cells in the logarithmic growth phase underwent ongoing irradiation at the dose of 1 Gy three times, 2 Gy three times, and 4 Gy seven times. Finally, cells were screened designated as radioresistant KYSE30 (named KYSE30R in figures), and were preserved in liquid nitrogen.

### In vitro* cell irradiation experiments*

Cells in each group were cultured in disposable T25 culture flasks at a density of about 5 × 10^6^ cells/flask and placed in an incubator with 5% CO_2_ at 37 ºC for 16 h. Before irradiation, the flask was filled with culture medium, and the condensate plate was used as a medium of 1.5 cm thickness. Irradiation of cells was conducted using a medical electron linear accelerator set to deliver a total dose of 8 Gy at a rate of 5 Gy/min at a 100 cm source axis distance. The cells were further cultured for 48 h after irradiation.

### RNA isolation and quantitation

Total RNA was extracted using Trizol (15,596,026, Invitrogen). Next, total RNA was reversely transcribed into cDNA according to the instructions of PrimeScript RT reagent Kit (RR047A, Takara Bio Inc., Otsu, Shiga, Japan). The RNA was reversely-transcribed into cDNA for miRNA detection using miRNA First Strand cDNA Synthesis (Tailing Reaction) kit (B532451-0020, Shanghai Sangon Biotechnology Co., Ltd., Shanghai, China). The reverse transcription quantitative polymerase chain reaction (RT-qPCR) was conducted for synthetized cDNA using Fast SYBR Green PCR kit (Applied Biosystems, Carlsbad, CA) and ABI7500 qPCR instrument (ABI Company, Oyster Bay, NY). GAPDH and U6 were regarded as the internal reference to quantify relative expression using the 2^−ΔΔCt^ method (Additional file 1: Table S1).

### Western blot analysis

The total protein was extracted from cells and tissues using radio-immunoprecipitation assay cell lysis buffer (BOSTER Biological Technology Co., Ltd., Wuhan, Hubei, China). The protein samples were separated by 10% SDS-PAGE, and transferred onto a PVDF membrane. The membrane was blocked for 1 h, and incubated overnight at 4ºC with the rabbit anti-human primary antibodies HDAC4 (ab111318, 1 μg/mL, 1: 500), HDAC2 (ab32117, 1: 2000), HDAC6 (ab133493, 1: 10,000), HDAC8 (ab187139, 1: 5000), HDAC1 (ab109411, 1: 1000), HDAC9 (ab109446, 1: 5000), pro caspase3 (ab32499, 1: 5000), cleaved-caspase3 (ab49822, 1: 500), pro caspase8 (ab108333, 1: 1000), caspase 8 (ab25901, 1 μg/mL, 1: 500), and GAPDH (ab181602, 1:5000, internal reference). The abovementioned antibodies were from Abcam Inc. (Cambridge, UK). Next, the membrane was supplemented with horseradish peroxidase-labeled goat anti-rabbit immunoglobulin G secondary antibody (ab205718, 1: 10,000, Abcam) for 1-h incubation, followed by visualization with the enhanced chemiluminescence. Finally, images were obtained with Bio-Rad image analysis system SmartView Pro 2000 (UVCI-2100, Major Science, CA), and the Quantity One software was employed for quantitative analysis of protein.

### Flow cytometry based Annexin V-fluorescein isothiocyanate/propidium iodide (FITC/PI) apoptosis assay

Cells were collected and centrifuged at 2000 rpm for 5 min, and the medium was discarded. Cells were suspended in 400 μL 1 × Binding Buffer and incubated with 5 μL Annexin V-FITC at 4 °C for 15 min in the dark. Subsequently, cells were cultured with 10 μL PI at 4 °C for 5 min in the dark. The cell cycle and apoptosis were detected using flow cytometer (BD FACS Calibur, BD Biosciences, Franklin Lakes, NJ) within 1 h.

### Colony formation assay

The 6-well plates were added with 2 mL 0.6% agar (Gibco). After solidification, cells were resuspended in 0.3% agar at 37 °C, and this cell suspension was added into 6-well plates containing agar (2000 cells/well). After further incubation at 37 °C for 14 days, the formed colonies were counted under the microscope (cell numbers ≥ 50% were regarded as one colony).

### Dual luciferase reporter gene assay

Cells were seeded into the 6-well plates at a density of 2 × 10^5^ cells/well. After cell adherence, cells were transfected according to the above methods. After successful transfection, cells were cultured for 48 h and collected. The luciferase activity of IRAK1 was detected according to the instructions of Dual Luciferase Reporter Gene Assay Kit (D0010, Beijing Solarbio Science & Technology Co., Ltd., Beijing, China). The luciferase intensity of IRAK1 was detected using Glomax20/20 luminometer fluorescence detector (E5311, Shanxi Zhongmei Biotechnology Co., Ltd., Xi’an, China).

### Immunohistochemistry

The tissue sections were subjected to antigen retrieval in citrate buffer. Then, sections were incubated with 3% H_2_O_2_ containing 0.3% Triton. Sections were then blocked with 10% goat serum for 60 min, incubated with primary antibody overnight at 4 °C and allowed to bind with secondary antibody according to manual of ElivisionTMsuper reagent kit. After addition of diaminobezidin, staining was observed under a microscope. Following a drop of hematoxylin, sections were added with lithium carbonate to return blue in color. The percentage of HDAC4 positive cells was calculated in 5 fields on the diagonal line of each section using five-point sampling method.

### Tumorigenicity assay in nude mice

A total of 36 BALB/c nude mice with specific pathogen free (SPF) grade (aged 3—4 weeks and weighing 18—20 g) were purchased from Shanghai SLAC Laboratory Animal Co., Ltd. (Shanghai, China). The mice were randomly divided into 6 groups (n = 6) and subjected to inoculation of cell suspensions which were prepared as follows: a total of 6 × 10^6^ radioresistant KYSE30 cells were transduced with plasmids containing shRNA targeting HDAC4 or IRAK1, or negative control (NC) shRNA. The cells were then suspended, and cell suspensions were mixed with Matrigel at a volume ratio of 1: 1 and inoculated subcutaneously into the nude mice. About two weeks later when the tumors had grown to about 0.5 cm^3^ in size, the mice were unirradiated or treated with local X-ray irradiation (8 Gy) at 2 Gy four times at two-day intervals. The length (L) and width (W) of the tumors were recorded. At the 25^th^ day, mice were euthanized by intraperitoneal barbiturate overdose, and the tumors were excised and weighed.

### Statistical analysis

All measurement data were expressed as mean ± standard deviation, as analyzed by SPSS 21.0 software (IBM Corp., Armonk, NY). Comparisons between two groups for normally distributed unpaired data were carried out using unpaired *t*-test, while those for data with skew distribution using Mann–Whitney *t*-test. Differences among multiple groups were analyzed by one-way analysis of variance (ANOVA), followed by Tukey’s post hoc test. Variables at different time points were tested by two-way ANOVA, followed by Bonferroni post hoc test. Survival data for patients were analyzed by the Kaplan–Meier method with the Log-rank test. Statistical significance was set at *p* < 0.05.

## Results

### HDAC4 was upregulated in radioresistant EC tissues and cells

**Fig. 1:**
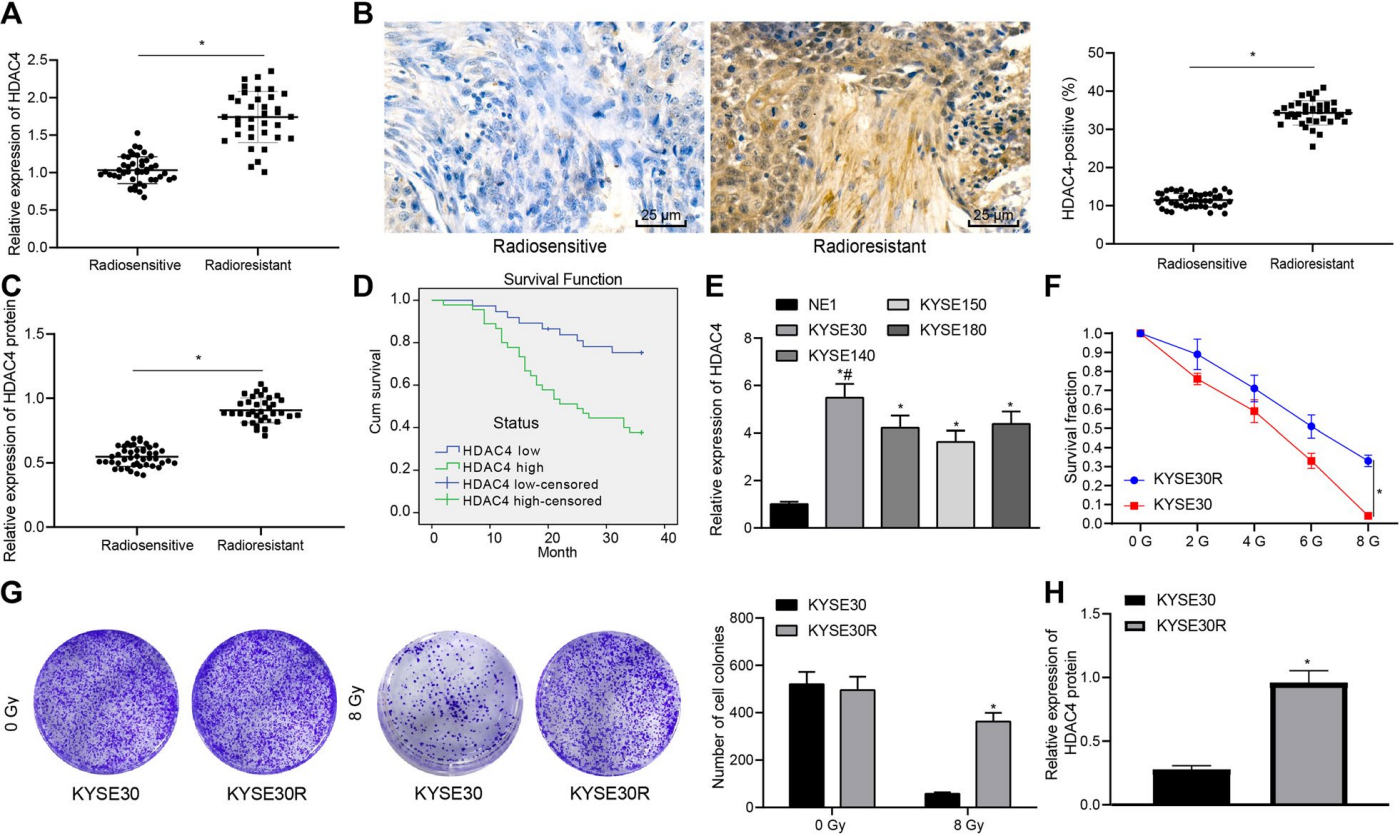
HDAC4 is highly expressed in radioresistant EC tissues and cells. **A** HDAC4 mRNA level in radioresistant (n = 46) and radiosensitive ESCC (n = 36) tissues was determined using RT-qPCR, normalized to GAPDH. **B** HDAC4 expression in radioresistant (n = 46) and radiosensitive ESCC (n = 36) tissues was determined using immunohistochemistry. **C** HDAC4 protein level in radioresistant (n = 46) and radiosensitive ESCC (n = 36) tissues was determined using Western blot analysis, normalized to GAPDH. **D** The Kaplan–Meier curve was used to plot the OS of patients according to median value of HDAC4 mRNA expression (> 1.218, high HDAC4 expression; < 1.218, low HDAC4 expression); low censored indicates missing data points of patients with low HDAC4 expression due to personal factors, while high censored indicates missing data points of patients with high HDAC4 expression due to personal factors. **E** HDAC4 mRNA level in normal esophageal epithelial cell line and EC cell lines was determined using RT-qPCR, normalized to GAPDH. **F** The survival of radioresistant KYSE30 cells and parental cells under X-ray irradiation of 2, 4, 6, and 8 Gy was detected by colony formation assay. **G** Colony-formation ability of radioresistant KYSE30 (KYSE30R) cells and parental cells under 0 or 8 Gy X-ray irradiation was detected by colony formation assay and statics of colonies. **H** HDAC4 expression in radioresistant KYSE30 (KYSE30R) cells and parental cells was determined using Western blot analysis. Values obtained from three independent experiments in triplicate are expressed as mean ± standard deviation and analyzed by Mann–Whitney *t* test. Values between two groups were analyzed by unpaired *t*-test, values among three or more groups by one-way ANOVA followed by Tukey's post hoc test, and effects as a function of irradiation dose were analyzed by two-way ANOVA followed by Bonferroni post hoc test. * *p* < 0.05 compared with radiosensitive ESCC tissues, NE1, or KYSE30 cells. ^#^*p* < 0.05 compared with KYSE140, KYSE150, and KYSE180 cells

We used RT-qPCR, immunohistochemistry, and Western blot analysis to determine the HDAC4 expression in radioresistant and radiosensitive ESCC tissues, results of which showed that HDAC4 expression conspicuously increased in radioresistant ESCC tissues (Fig. 1A–C). Kaplan–Meier analysis revealed that the overall survival (OS) of patients with high HDAC4 expression was lower (Fig. 1D). Moreover, RT-qPCR displayed increased HDAC4 expression in the EC cell lines KYSE30, KYSE140, KYSE150, and KYSE180. Among these, we selected the KYSE30 cell line with highest HDAC4 expression (Fig. 1E), for construction of our radioresistant cell line.

Compared with KYSE30 cells, the survival function of radioresistant KYSE30 cells increased under X-ray irradiation at doses of 2, 4, 6, and 8 Gy (Fig. 1F), showing that radioresistant KYSE30 cells were indeed more resistant to radiation. The colony formation assay displayed no significant difference in clonogenic potential between radioresistant KYSE30 cells and parental cells under X-ray irradiation at a dose of 0 Gy (*p* > 0.05), while under X-ray irradiation at a dose of 8 Gy, the clonogenic potential of radioresistant KYSE30 cells was higher than that of parental cells (Fig. 1G). Western blot analysis confirmed that HDAC4 expression was higher in radioresistant KYSE30 cells than that in parental cells (Fig. 1H, Additional file 2: Figure S1A). In addition, protein levels of HDAC1, HDAC2, HDAC6, HDAC8 and HDAC9 in radioresistant KYSE30 cells, revealing slight increase of HDAC2 and HDAC6 expression in radioresistant KYSE30 cells than that in parental cells (Additional file 3: Figure S2).

Overall, these results suggested that HDAC4 expression in radioresistant EC tissues and cells, and that this elevation influenced the prognosis of patients with ESCC.

### Downregulation of HDAC4 promoted radiosensitivity of EC cells

**Fig. 2:**
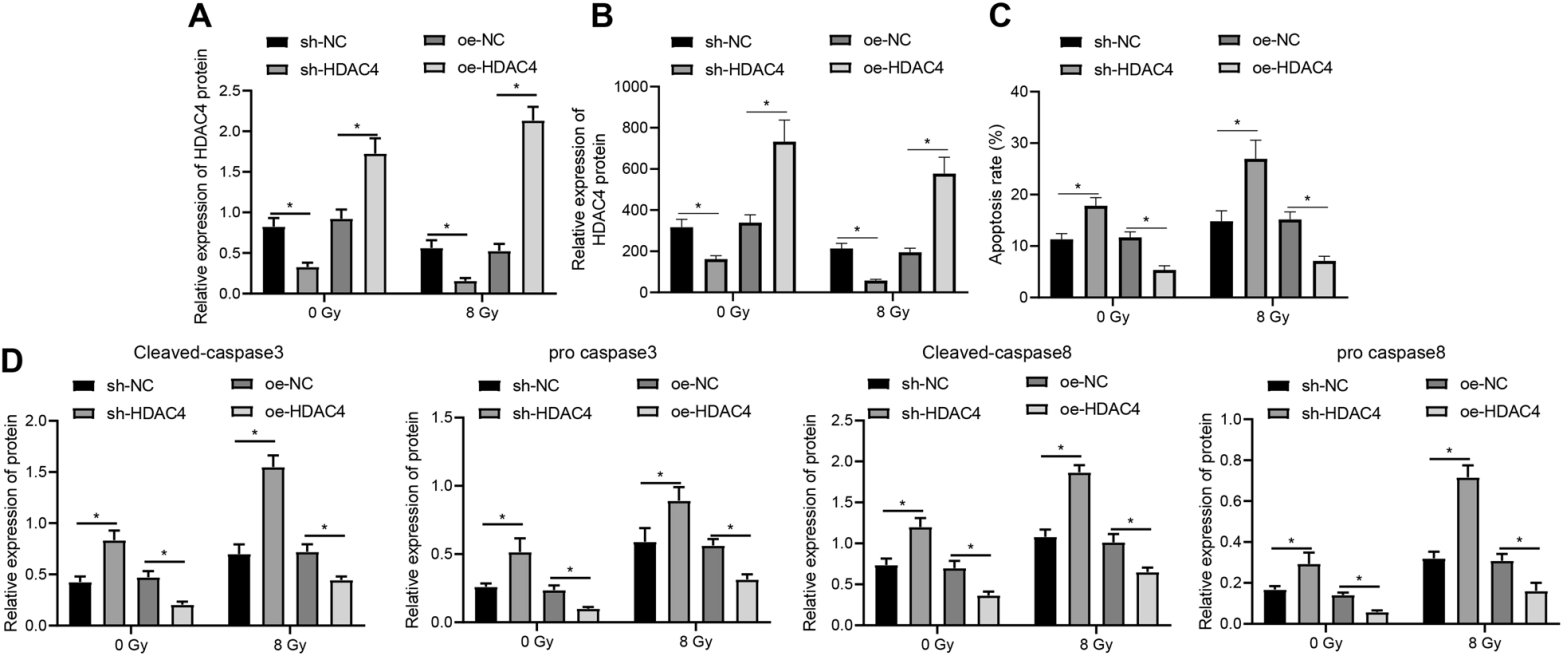
Silencing of HDAC4 induces apoptosis and radiosensitivity of EC cells. Radioresistant KYSE30 (KYSE30R) cells were transduced with HDAC4 overexpression (oe-) plasmids or plasmids containing shRNA (sh-) targeting HDAC4 under X-ray (8 Gy) or under no irradiation (0 Gy). **A** HDAC4 overexpression and knockdown in radioresistant KYSE30 cells was verified using Western blot analysis, normalized to GAPDH. **B** Colony-formation ability of radioresistant KYSE30 cells was detected by colony formation assay in response to HDAC4 overexpression and knockdown. **C** Apoptosis of radioresistant KYSE30 cells was detected by flow cytometry based Annexin V-FITC/PI apoptosis assay in response to HDAC4 overexpression and knockdown. **D** Expression of cleaved-caspase3, cleaved-caspase8, pro caspase3 and pro caspase8 in radioresistant KYSE30 cells with HDAC4 overexpression and knockdown was measured using Western blot analysis, normalized to GAPDH. Values obtained from three independent experiments in triplicate were expressed as mean ± standard deviation and values between two groups were analyzed by unpaired *t*-test. * *p* < 0.05

After identifying the upregulation of HDAC4 in radioresistant EC tissues and cells, we then managed to explore the effect of HDAC4 on radiotherapy resistance of EC cells. First, we employed Western blot analysis to validate the knockdown efficiency of shRNA targeting HDAC4 (Additional file 4: Figure S3A), identifying the optimal sh-HDAC4-2 for following experiments. Radioresistant KYSE30 cells were then treated with overexpressed or silenced HDAC4 under X-ray irradiation at a dose of 8 Gy, and Western blot analysis displayed that HDAC4 expression was decreased in response to sh-HDAC4 and augmented in response to oe-HDAC4 (Fig. 2A, Additional file 2: Figure S1B).

Colony formation assay revealed that silencing of HDAC4 inhibited, and HDAC4 overexpression facilitated, the colony formation of unirradiated or irradiated EC cells, whereas irradiation treatment (8 Gy) led to suppressed clonogenic potential of the cells as compared with untreated ones (Fig. 2B). In addition, flow cytometry and Western blot analysis demonstrated that HDAC4 knockdown resulted in accelerated apoptotic potential as well as elevated levels of cleaved-caspase3 and cleaved-caspase8, and HDAC4 overexpression led to the opposite results; moreover, the expression of cleaved-caspase3/8 was elevated in the presence of irradiation (Fig. 2C, D).

Therefore, it could be concluded that HDAC4 knockdown induced apoptotic potential of and radiosensitized EC cells.

### HDAC4 inhibited radiosensitivity of EC cells by inhibiting miR-146a expression

**Fig. 3:**
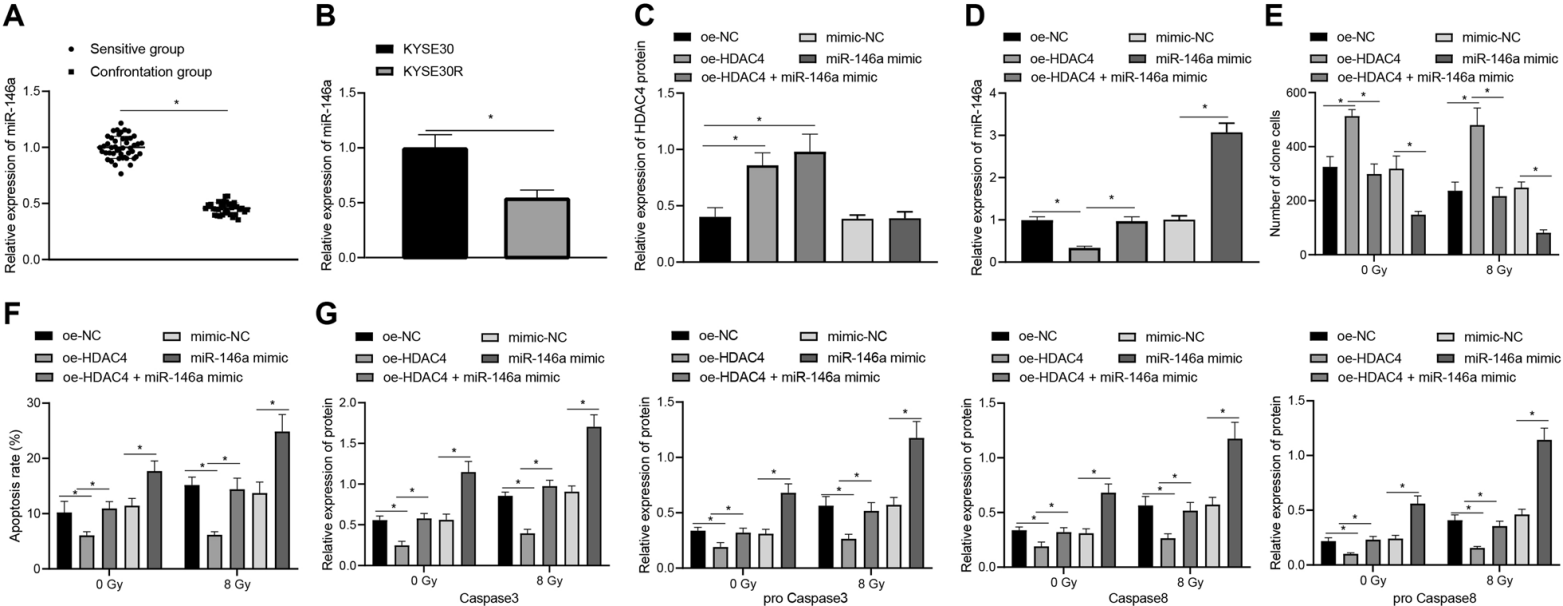
HDAC4 enhances radioresistance of EC cells via downregulation of miR-146a. Radioresistant KYSE30 (KYSE30R) cells were treated with exogenous miR-146a mimic and expression vectors (oe-) containing the HDAC4 alone or in combination under 8 Gy X-ray irradiation. **A** miR-146a expression in radioresistant (n = 46) and radiosensitive ESCC (n = 36) tissues was determined using RT-qPCR, normalized to U6. **B** miR-146a expression in radioresistant KYSE30 cells and parental cells was determined using RT-qPCR, normalized to U6. **C** HDAC4 protein level in radioresistant KYSE30 cells was determined using Western blot analysis, normalized to GAPDH. **D** miR-146a expression in radioresistant KYSE30 cells was determined using RT-qPCR, normalized to U6. **E** Colony-formation ability of irradiated (8 Gy)/unirradiated (0 Gy) radioresistant KYSE30 cells was detected by colony formation assay. **F** Apoptosis of irradiated (8 Gy)/unirradiated (0 Gy) radioresistant KYSE30 cells was detected by flow cytometry based Annexin V-FITC/PI apoptosis assay. **G** Expression of cleaved-caspase3, cleaved-caspase8, pro caspase3 and pro caspase8 in irradiated (8 Gy)/unirradiated (0 Gy) radioresistant KYSE30 cells was measured using Western blot analysis, normalized to GAPDH. Values obtained from three independent experiments in triplicate were expressed as mean ± standard deviation. Values between two groups were analyzed by unpaired *t*-test, and values among three or more groups by one-way ANOVA followed by Tukey's post hoc test. * *p* < 0.05

Since the aforementioned results revealed the suppressing of HDAC4 imparting radiosensitivity of EC cells, RT-qPCR showed reduced miR-146a expression in radioresistant ESCC tissues and in radioresistant KYSE30 cells (Fig. 3A, B).

To understand the effects of HDAC4 and miR-146a on radioresistance of EC, radioresistant KYSE30 cells were treated with overexpressed HDAC4 and exogenous miR-146a mimic and expression vectors containing the HDAC4 in combination, respectively, under X-ray irradiation at a dose of 0/8 Gy. According to Western blot analysis, HDAC4 expression was increased in radioresistant KYSE30 cells treated with plasmids overexpressing HDAC4, and the combination of HDAC4 overexpression and miR-146a mimic witnessed no obvious change in HDAC4 expression as compared with HDAC4 overexpression alone (Fig. 3C). Meanwhile, the expression of miR-146a, as measured by RT-qPCR, was downregulated in the presence of HDAC4 overexpression alone, and this downregulation was negated by the combination of HDAC4 and miR-146a overexpression (Fig. 3D).

It was revealed that HDAC4 overexpression promoted the colony formation ability of EC cells, whereas such promotion was repressed when HDAC4 overexpression was combined with miR-146a overexpression (Fig. 3E).

Consistently, the inhibitory effect of HDAC4 overexpression on the apoptotic potential of EC cells, as reflected by flow cytometry and Western blot determination of cleaved-caspase3/8 and pro caspase3/8, was minimized by its combination with miR-146a overexpression (Fig. 3F, G). Additionally, the aforementioned EC cells exhibited reduced clonogenic potential and enhanced apoptotic ability when subjected to irradiation at a dose of 8 Gy as compared with their unirradiated counterparts.

Taken together, HDAC4 inhibited radiosensitivity of EC cells by inhibiting miR-146a expression.

### IRAK1 was upregulated in radioresistant EC tissues and cells and targeted by miR-146a

**Fig. 4:**
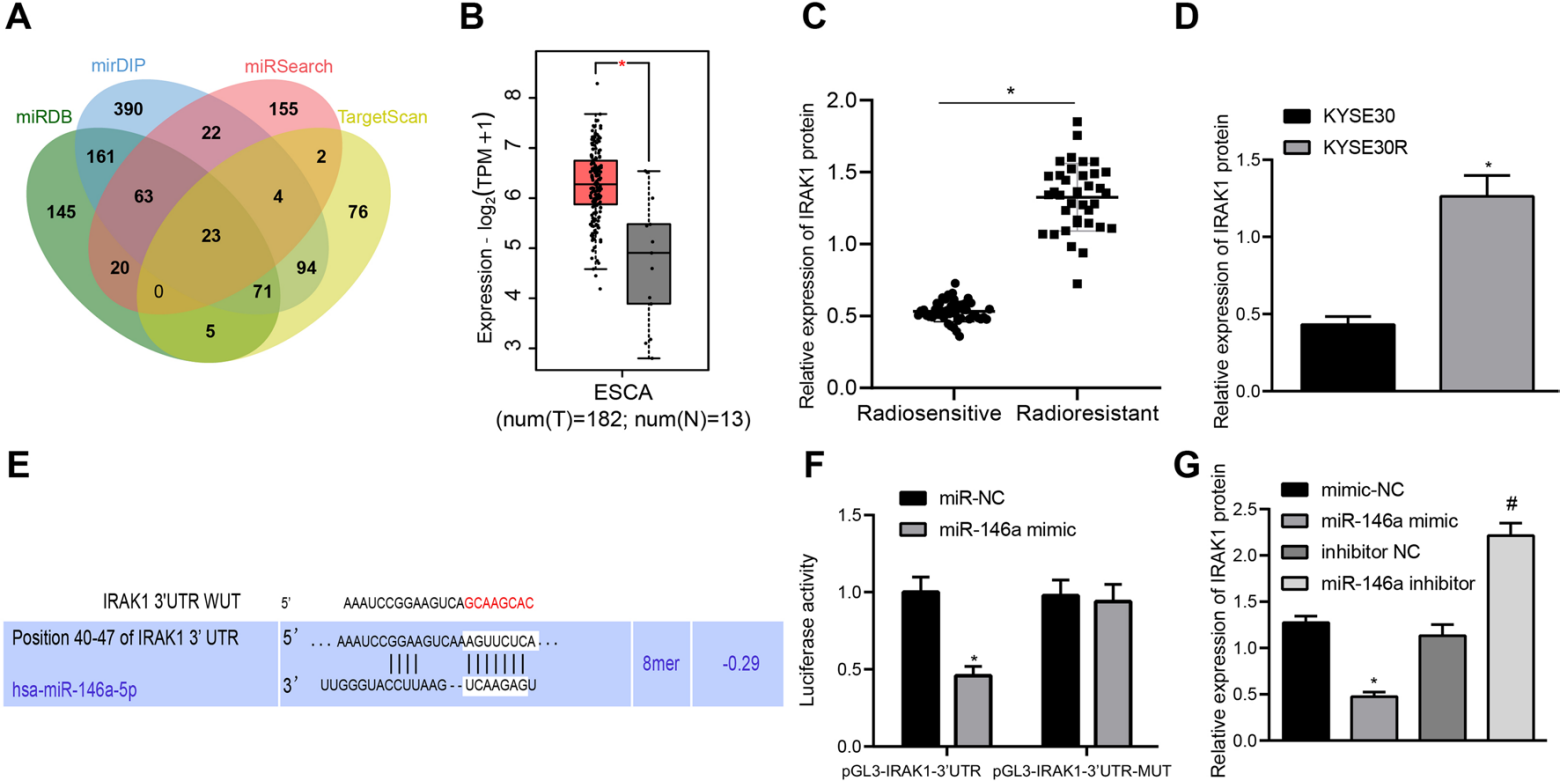
IRAK1 is highly expressed in radioresistant EC tissues and cells and is targeted by miR-146a. **A** Target genes of miR-146a predicted using microDB, mirDIP, and other databases. The five ellipses in the graph represent the prediction results of five databases, and the middle part represented the Venn intersection of the prediction results in five databases. **B** Analysis of IRAK1 expression in ESCC in TCGA database. The abscissa refers to the type of sample, and the ordinate is the level of gene expression. The red box represents the tumor sample, and the gray box represents the normal sample. * *p* < 0.01. **C** IRAK1 expression in radioresistant (n = 46) and radiosensitive ESCC (n = 36) tissues was determined using Western blot analysis, normalized to GAPDH. **D** IRAK1 expression in KYSE30 and radioresistant KYSE30 (KYSE30R) cells was determined using Western blot analysis, normalized to GAPDH. **E** Putative miR-146a binding sites in the IRAK1 mRNA 3’UTR. **F** IRAK1 as the target of miR-146a was evaluated using dual-luciferase reporter gene assay. **G** IRAK1 expression in radioresistant KYSE30 cells with elevated miR-146a was determined using Western blot analysis, normalized to GAPDH. Values obtained from three independent experiments in triplicate were expressed as mean ± standard deviation. Values between two groups were analyzed by unpaired *t*-test. * *p* < 0.05 compared with radiosensitive ESCC tissues, KYSE30 cells, or radioresistant KYSE30 cells treated with mimic NC. ^#^*p* < 0.05 compared with radioresistant KYSE30 cells treated with inhibitor NC

Following the HDAC4/miR-146a axis, we further explored the downstream targets of miR-146a and 23 candidate genes were predicted using microDB, mirDIP, and other databases (Fig. 4A). IRAK1 expression was further analyzed in EC in the TCGA database (Fig. 4B), which revealed elevated IRAK1 expression.

Moreover, Western blot analysis showed increased IRAK1 expression in radioresistant ESCC tissues of both radiosensitive (n = 46) and radioresistant (n = 36) patients (Fig. 4C), and also in radioresistant KYSE30 cells (Fig. 4D, Additional file 2: Figure S1C). The online database targetscan predicted that there was a specific binding region between miR-146a and IRAK1 (Fig. 4E). Dual-luciferase reporter gene assay verified that luciferase activity of IRAK1 3’untranslated region (3’UTR)-wild type rather than that of IRAK1 3’UTR-mutant was reduced in the presence of miR-146a mimic (Fig. 4F). In addition, decreased IRAK1 expression was observed in radioresistant KYSE30 cells after overexpression of miR-146a by its specific mimic, while miR-146a knockdown led to the opposite results (Fig. 4G, Additional file 2: Figure S1D).

In summary, IRAK1, upregulated in radioresistant EC tissues and cells, was a target gene of miR-146a.

### Silencing of IRAK1 promoted radiosensitivity of EC KYSE30 cells

**Fig. 5:**
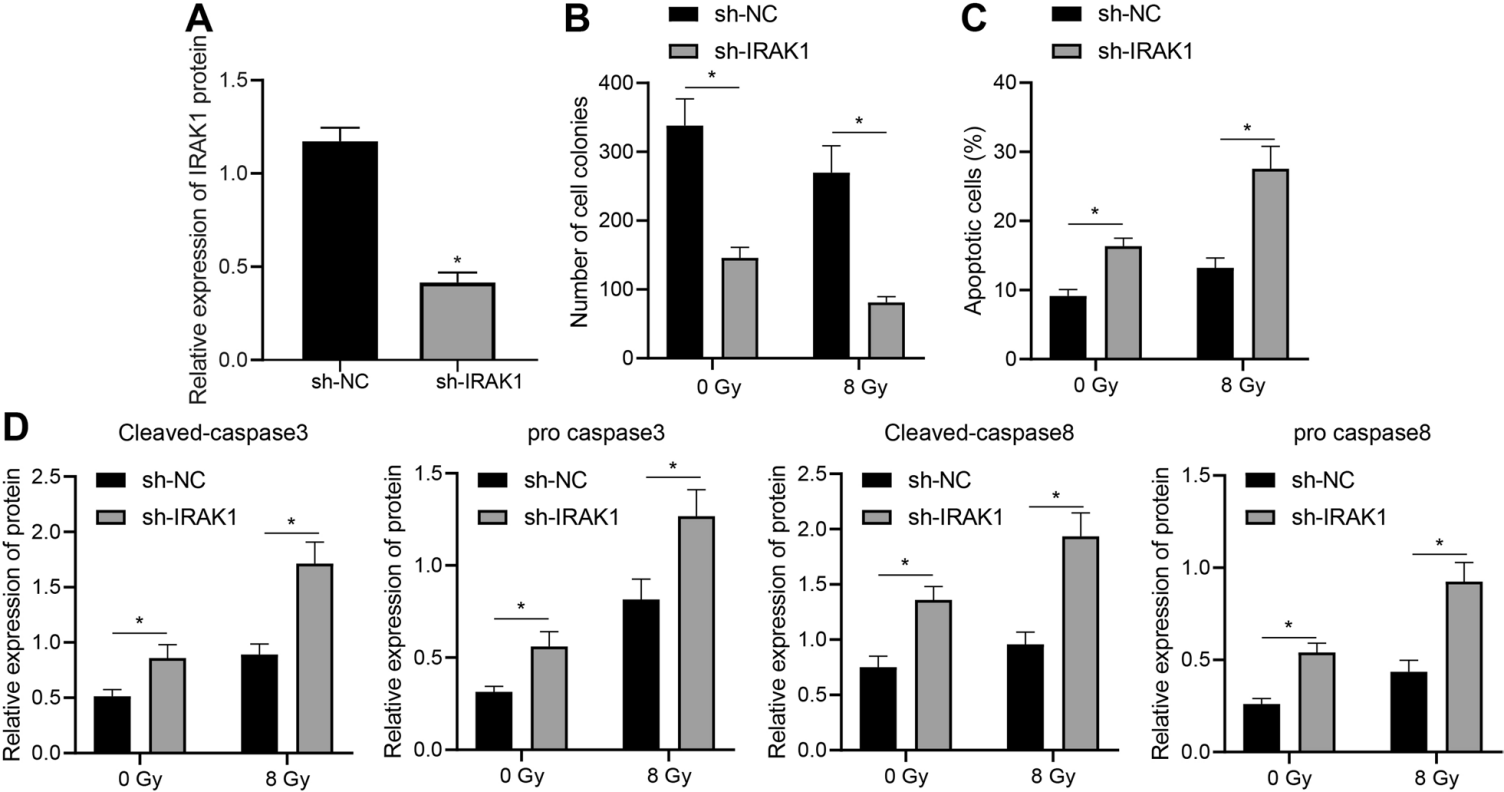
Knockdown of IRAK1 induces apoptosis of and radiosensitized EC KYSE30 cells. The radioresistant KYSE30 (KYSE30R) cell line was treated with sh-IRAK1. **A** IRAK1 knockdown in radioresistant KYSE30 cells was determined using Western blot analysis, normalized to GAPDH. **B** Colony-formation ability of irradiated (8 Gy)/unirradiated (0 Gy) radioresistant KYSE30 cells with IRAK1 knockdown under 0/8 Gy X-ray irradiation was detected by colony formation assay. **C** Apoptosis of radioresistant KYSE30 cells with IRAK1 knockdown under 8 Gy X-ray or under no irradiation detected by flow cytometry based Annexin V-FITC/PI apoptosis assay. **D** Expression of cleaved-caspase3, cleaved-caspase8, pro caspase3 and pro caspase8 in radioresistant KYSE30 cells with IRAK1 knockdown under 8 Gy X-ray or under no irradiation measured using Western blot analysis, normalized to GAPDH. Values obtained from three independent experiments in triplicate were expressed as mean ± standard deviation. Values between two groups were analyzed by unpaired *t*-test. * *p* < 0.05

Furthermore, IRAK1-silencing radioresistant KYSE30 cells were constructed under X-ray irradiation at a dose of 0/8 Gy to verify the effect of IRAK1 on radioresistance of EC cells. Based on Western blot analysis results, sh-IRAK1-2 exhibited best knockdown efficiency and was thus selected for subsequent experiments (Additional file 4: Figure S3B), and downregulated expression of IRAK1 was identified in sh-IRAK1-2-treated KYSE30 cells (Fig. 5A, Additional file 2: Figure S1E).

Colony formation assay revealed that silencing of IRAK1 inhibited colony formation ability of the KYSE30 cells (Fig. 5B). In addition, flow cytometry and Western blot analysis substantiated that IRAK1 knockdown elevated apoptotic potential and levels of cleaved-caspase3 and cleaved-caspase8 in KYSE30 cells, and the levels of pro caspase3/8 didn’t differ significantly (Fig. 5C, D). Moreover, the aforementioned effects of silencing IRAK1 were unacted on the presence of irradiation, while irradiation treatment (8 Gy) resulted in reduced clonogenic potential and enhanced apoptotic ability of the cells versus their unirradiated counterparts. Collectively, these results revealed that IRAK1 silencing triggered apoptotic potential and promoted radiosensitivity of EC KYSE30 cells.

### HDAC4 inhibited radiosensitivity of EC cells via the miR-146a/IRAK1 axis

**Fig. 6:**
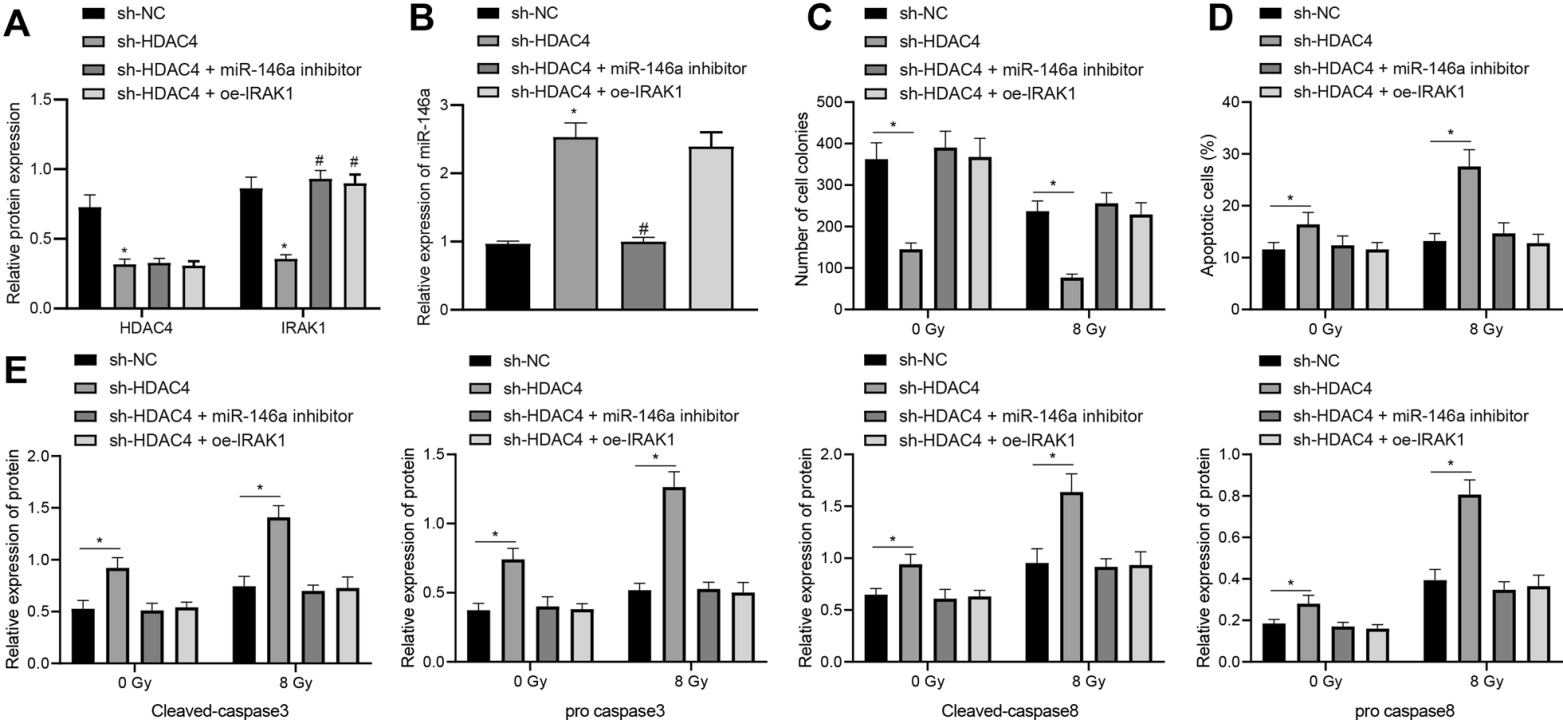
Knockdown of HDAC4 radiosensitizes EC KYSE30 cells via upregulation of miR-146a and downregulation of IRAK1. Radioresistant KYSE30 (KYSE30R) cells were treated with shRNA (sh-) against HDAC4 and exogenous miR-146a inhibitor alone or in combination under 8 Gy X-ray irradiation. **A** HDAC4 and IRAK1 expression in radioresistant KYSE30 cells was determined using Western blot analysis, normalized to GAPDH. **B** miR-146a expression in radioresistant KYSE30 cells was determined using RT-qPCR, normalized to U6. **C** Colony-formation ability of irradiated (8 Gy)/unirradiated (0 Gy) radioresistant KYSE30 cells was detected by colony formation assay. **D** Apoptosis of irradiated (8 Gy)/unirradiated (0 Gy) radioresistant KYSE30 cells was detected by flow cytometry based Annexin V-FITC/PI apoptosis assay. **E** Expression of cleaved-caspase3, cleaved-caspase8, pro caspase3 and pro caspase8 in irradiated (8 Gy)/unirradiated (0 Gy) radioresistant KYSE30 cells was measured using Western blot analysis, normalized to GAPDH. Values obtained from three independent experiments in triplicate were expressed as mean ± standard deviation. Values among three or more groups by one-way ANOVA followed by Tukey's post hoc test. * *p* < 0.05 compared with irradiated (8 Gy)/unirradiated (0 Gy) radioresistant KYSE30 cells treated with scramble shRNA. ^#^*p* < 0.05 compared with irradiated (8 Gy)/unirradiated (0 Gy) radioresistant KYSE30 cells treated with sh-HDAC4

Next, we managed to validate the regulation mechanism of the HDAC4/miR-146a/IRAK1 axis in EC cells by manipulating their expression in KYSE30 cells with/without irradiation (0/8 Gy). Western blot analysis displayed that HDAC4 and IRAK1 expression was decreased in radioresistant KYSE30 cells in response to HDAC4 knockdown alone, and IRAK1 expression was increased while HDAC4 expression was unaffected in radioresistant KYSE30 cells in response to the combination of HDAC4 knockdown and miR-146a inhibition/IRAK1 overexpression (Fig. 6A, Additional file 2: Figure S1F). RT-qPCR showed elevated miR-146a expression in radioresistant KYSE30 cells in the presence of HDAC4 knockdown, while this elevation was negated when HDAC4 knockdown was combined with miR-146a inhibition (Fig. 6B).

Further, the inhibitory effect of HDAC4 knockdown on the colony formation ability of KYSE30 cells as well as its cell apoptosis-promoting effect (increased cleaved-caspase3/8, unchanged pro caspase3/8) were both mitigated when it was combined with miR-146a suppression or IRAK1 overexpression (Fig. 6C–E). Taken together, these findings illuminated that HDAC4 silencing attenuated the radioresistance of EC cells by elevating miR-146a and suppressing IRAK1 expression.

### Silencing of HDAC4 or IRAK1 promoted radiosensitivity of EC cells in vivo

**Fig. 7:**
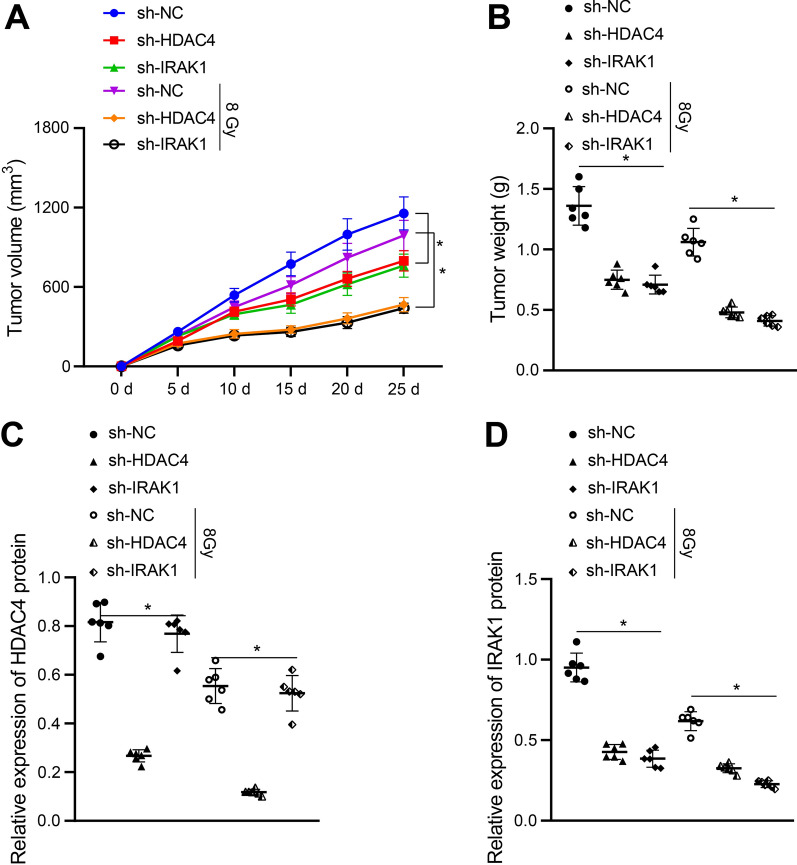
Knockdown of HDAC4 or IRAK1 promotes radiosensitivity of EC cells in vivo. Nude mice were subcutaneously injected with radioresistant KYSE30 (KYSE30R) cells with shRNA (sh-) against HDAC4 or IRAK1. These mice were unirradiated or received local X-ray irradiation at a dose of 8 Gy. **A** Tumor volume in nude mice. **B** Tumor weight in nude mice. **C** HDAC4 protein level in tumor tissues was measured by using Western blot analysis, normalized to GAPDH. **D** IRAK1 protein level in tumor tissues was measured by using Western blot analysis, normalized to GAPDH. Values obtained from three independent experiments in triplicate were expressed as mean ± standard deviation. Values among three or more groups were compared by one-way ANOVA followed by Tukey's post hoc test, and results at different time points were compared by two-way ANOVA followed by Bonferroni’s post hoc test. * *p* < 0.05. Each group consists of 6 mice

Following the in vitro experiments demonstrating the inhibitory action of HDAC4 on radiosensitivity in radioresistant KYSE30 cells via the miR-146a/IRAK1 axis, we then moved to in vivo substantiation of the aforementioned findings. Nude mice with subcutaneous xenografted tumors were subjected to radiotherapy (0/8 Gy), followed by collection of tumors and images. As a result, tumor volume and weight were found to be reduced in the presence of HDAC4 or IRAK1 knockdown either under or not under irradiation, while the reduction was more substantial in the presence of irradiation (8 Gy) (Fig. 7A, B, Additional file 5: Figure S4). Further Western blot analysis showed that HDAC4 expression was decreased in the tumors after silencing of HDAC4, but was unaffected after silencing of IRAK1 (*p* > 0.05) while HDAC4 protein level was diminished in tumors under irradiation at a dose of 8 Gy when compared with those without irradiation (Fig. 7C). Furthermore, Western blot analysis (Fig. 7D) showed decreased IRAK1 expression in response to silencing of HDAC4 or IRAK1. In addition, the protein expression of IRAK1 was reduced in tumors exposed to 8 Gy irradiation, relative to 0 Gy irradiation. Collectively, our data established that silencing of HDAC4 or IRAK1 promoted radiosensitivity of EC cells in vivo.

## Discussion

**Fig. 8:**
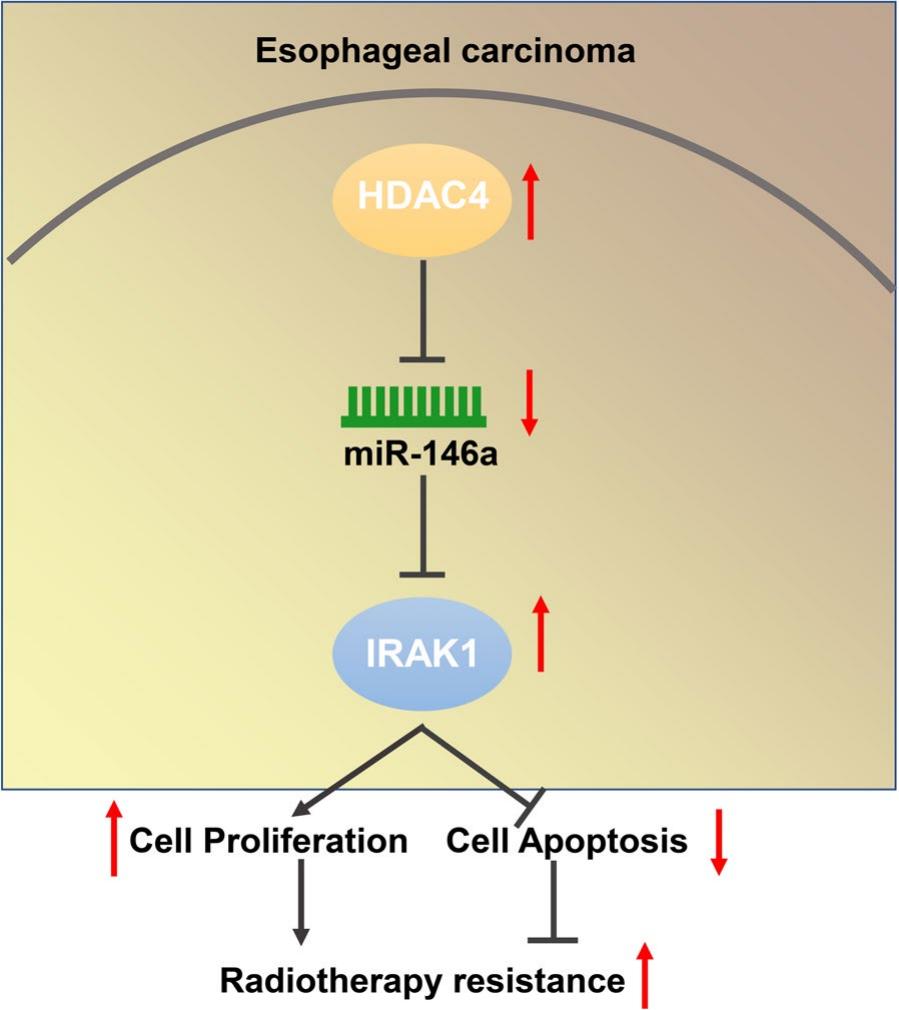
The mechanism graph of the regulatory network of HDAC4 in EC. HDAC4 overexpression occurs in radioresistant EC cells. HDAC4 promotes cell proliferation and inhibits apoptosis of EC cells by downregulating miR-146a expression and elevating IRAK1 expression, leading to promoted radioresistance of EC cells

Radiotherapy is the most effective treatment for EC, a dangerous tumor with high morbidity and mortality. Nonetheless, many patients with EC develop tumor recurrence or metastasis due to development of radioresistance [16]. Evidence accumulating in recent years implicates the significance of HDACs in the progression of EC [17]. Moreover, a recent study has demonstrated that miRNAs play an important role in irradiation-induced apoptosis and the onset of radioresistance of tumor cells [18]. Therefore, we investigated the influence of HDAC4 regulating miR-146a expression on the radioresistance of EC cells (Fig. 8). Overall, our data elucidated that HDAC4 knockdown suppressed the clonogenic potential of EC cells and accelerated their apoptotic potential by modulating the miR-146a/IRAK1 axis, thereby promoting radiosensitivity of EC cells.

It was established in our study that HDAC4 was highly expressed in radioresistant EC tissues and cells. Indeed, dysregulation of HDACs has a close relationship with the progression of ESCC [5]. Also, Wang et al*.* have reported that HDAC2 is upregulated in ESCC tissues [19]. Moreover, we demonstrated that overexpressed HDAC4 was associated with unfavorable survival, as reflected by lower OS in patients with high HDAC4 expression. Our findings concur with another work showing that increased HDAC4 expression was witnessed in ESCC tissues and cell lines, and its upregulation aggravates EC progression and imparts poor survival [7]. These data further demonstrated the importance of investigating HDAC4 in radioresistant EC.

Other results in the present study confirmed the low expression of miR-146a in radioresistant EC tissues and cells. Previous works shows that aberrant miRNA expression plays a crucial role in radioresistance of ESCC cells [10]. Reduced miR-381 expression is reported in radiation-resistance ESCC tissues and cells [11], whereas downregulated miR-146a occurs in in head and neck squamous cell carcinoma [20]. Moreover, a bioinformatics website in combination with the dual luciferase reporter gene assay validated that miR-146a targeted and negatively regulated the expression of IRAK1, as likewise shown in a recent independent study [21]. A functional study has demonstrated high expression of IRAK1 in human tumor tissues, such as hepatocellular carcinoma and breast cancer [22]. These lines of evidence support the present finding that miR-146a and IRAK1 were downregulated in EC, and that miR-146a targeted and regulated IRAK1 expression.

Furthermore, the present results show that silencing of HDAC4 inhibited clonogenic potential and promoted radiosensitivity of EC cells and triggered apoptotic capacities by upregulating miR-146a expression and inhibiting IRAK1 expression in vitro and likewise in vivo. Accumulating evidence illustrates that silencing of HDAC radiosensitized EC cells. In this context, it is interesting to note that HDAC4 knockdown contributes to the inhibition of cell proliferative, migratory and apoptotic capacities in ESCC [7]. The induction of radioresistance by HDAC4 in glioblastoma has also been investigated [6]. Moreover, silencing of HDAC enhanced miR-146a expression, which suppressed IRAK1 expression via activation of the nuclear factor kappa-B pathway [8]. Overexpression of miR-146a suppresses gastric cancer cell migratory and invasive capacities by downregulating IRAK1 [23], whereas upregulated miR-146a-5p stimulates chemosensitivity of non-small cell lung cancer cells to cisplatin [24]. The elevation of miR-146a leads to suppression of cell proliferative and invasive capacities in ESCC, thus serving as an inhibitor in ESCC progression [25]. Restoration of miR-381 expression promoted radiosensitivity of ESCC cells and reduced tumor growth in vitro and in vivo by inhibiting cell proliferative and migratory capacities [11]. Meanwhile, downregulation of IRAK1 reduced paclitaxel resistance via facilitation of apoptotic ability in breast cancer [26]. Notably, a recent report has illuminated the additional conserved role of IRAK1 in the cell survival response to infrared rays and pointed out that IRAK1 contributed to intrinsic tumor resistance to radiotherapy [27]. These findings generally concur with the key finding of the current study, whereby silencing HDAC4 was found to augment miR-146a expression, inhibit IRAK1 expression, and thus suppress the clonogenic potential of EC cells and accelerate their apoptotic potential, thereby radiosensitizing EC cells. Our in vivo experiment further validated the inhibitory effect of HDAC4 knockdown on EC radioresistance.

More importantly, increasing evidence has documented that HDAC inhibitors improve radiosensibility in malignancies, including lung cancer, colon cancer, glioma and squamous cell carcinoma [28–31], suggesting the implication of HDACs in tumor radiation resistance. For instance, HDAC4 and HDAC6 have been indicated to make glioma cells resistant to radiation by maintaining DNA double-strand damage repair and stemness [6]. High expression of HDAC2 and HDAC8 has been demonstrated to be associated with resistance of breast cancer cells to radiation [32]. In the context of EC, highly expressed HDAC1 is found to be related to poor prognosis of patients [33]. Also, HDAC2, HDAC4 and HDAC9 are implicated in the growth and metastasis of EC cells as potential therapeutic targets [17, 34, 35]. Notably, HDAC inhibitors have been reported to reverse the acquired radioresistance of EC cells KYSE-150R [36]. In our study, it was found that HDAC2 and HDAC6 expression was slightly increased in radioresistant KYSE30 cells yet HDAC1, HDAC8 and HDAC9 expression insignificantly altered. Hereby, the significance of other members of HDAC family requires further investigation in the future.

## Conclusion

Taken together, the data acquired in the present study indicate that silencing of HDAC4 upregulated miR-146a, which may present a potential miR-targeted therapy for patients with radioresistant EC by enhancing cell apoptotic potential. Our findings elucidated the regulation mechanism of the HDAC4/miR-146a/IRAK1 axis on EC cells and shed light on this axis as a potential EC therapeutic target. Of note, HDAC4 inhibitors may be combined with radiotherapy in future clinical setting to kill EC cells yet the targeting drug delivery system requires urgent solutions considering the specific physiological position of EC. Also, this study is also limited by insufficient sample size, an extended investigation with a larger cohort of patients and biopsy samples from same stage of the disease involving multiple hospitals is required to be conducted in the future.

## Supplementary Information

**Additional file 1: Table S1.** Primer sequences for RT-qPCR

**Additional file 2: Figure S1** Representative Western blots for Fig. 1H (A), 2A (B), 4D (C), 4G (D), 5A (E), and 6A (F).

**Additional file 3: Figure S2** Expression of HDAC1, HDAC2, HDAC6, HDAC8 and HDAC9 in KYSE30 and radioresistant KYSE30 (KYSE30R) cell lines was determined by Western blot analysis. Values obtained from three independent experiments in triplicate were expressed as mean ± standard deviation. Values among three or more groups were compared by one-way ANOVA followed by Tukey's post hoc test. * *p* < 0.05 compared with KYSE30 cells.

**Additional file 4: Figure S3** Silencing efficiency of HDAC4 and IRAK1. (A) HDAC4 knockdown was determined by Western blot analysis, revealing sh-HDAC4-2 with best silencing efficiency. (B) IRAK1 knockdown was determined by Western blot analysis, revealing sh-IRAK1-3 with best silencing efficiency. Values obtained from three independent experiments in triplicate were expressed as mean ± standard deviation. Values among three or more groups were compared by one-way ANOVA followed by Tukey's post hoc test. * *p* < 0.05 compared with sh-NC. # *p* < 0.05 compared with sh-HDAC4-1 and sh-HDAC4-3 or sh-IRAK1-1 and sh-IRAK1-2.

**Additional file 5: Figure S4** Representative images of tumors collected from nude mice xenografted with radioresistant KYSE30 cells in response to sh-HDAC4 or sh-IRAK1. These mice were unirradiated or received local X-ray irradiation at a dose of 8 Gy.

## Data Availability

The datasets generated for this study are available on request to the corresponding author.

## Abbreviations

HDACs: Histone deacetylase
EC: Esophageal carcinoma
CR: Complete Remission
PR: Partial Remission
SD: Stable Disease
PD: Progressive Disease
RPMI: Roswell Park Memorial Institute
FBS: Fetal bovine serum
RIPA: Radio-immunoprecipitation assay
