# Supplementary material for: Promotion of microRNA-146a by histone deacetylase 4 silencing contributes to radiosensitization of esophageal carcinoma
Source: J Transl Med. 2022 Feb 22;20:101. doi: 10.1186/s12967-021-03171-z (PMC8862391; doi:10.1186/s12967-021-03171-z)
Supplement: Supplementary file 1 — Additional file 1: Table S1. Primer sequences for RT-qPCR [file 12967_2021_3171_MOESM1_ESM.docx]

**Table S1** Primer sequences for RT-qPCR

| Gene | Sequence (5'-3') | |
| --- | --- | --- |
| HDAC4 | Forward | GAGAGACTCACCCTTCCCG |
|  | Reverse | CCGGTCTGCACCAACCAAG |
| GAPDH | Forward | TGTGGGCATCAATGGATTTGG |
|  | Reverse | ACACCATGTATTCCGGGTCAAT |
| miR-146a | Forward  Reverse | TGAGAACTGAATTCCATGGGTT  CGACAGTTGCTATGCGATGCA |
| U6 | Forward | AAAGCAAATCATCGGACGACC |
|  | Reverse | GTACAACACATTGTTTCCTCGGA |

Note: RT-qPCR, reverse transcription quantitative polymerase chain reaction; HDAC4, histone deacetylase 4; GAPDH, glyceraldehyde-3-phosphate dehydrogenase; miR, microRNA.
