# Supplementary figures and images for: Promotion of microRNA-146a by histone deacetylase 4 silencing contributes to radiosensitization of esophageal carcinoma
Source: J Transl Med. 2022 Feb 22;20:101. doi: 10.1186/s12967-021-03171-z (PMC8862391; doi:10.1186/s12967-021-03171-z)

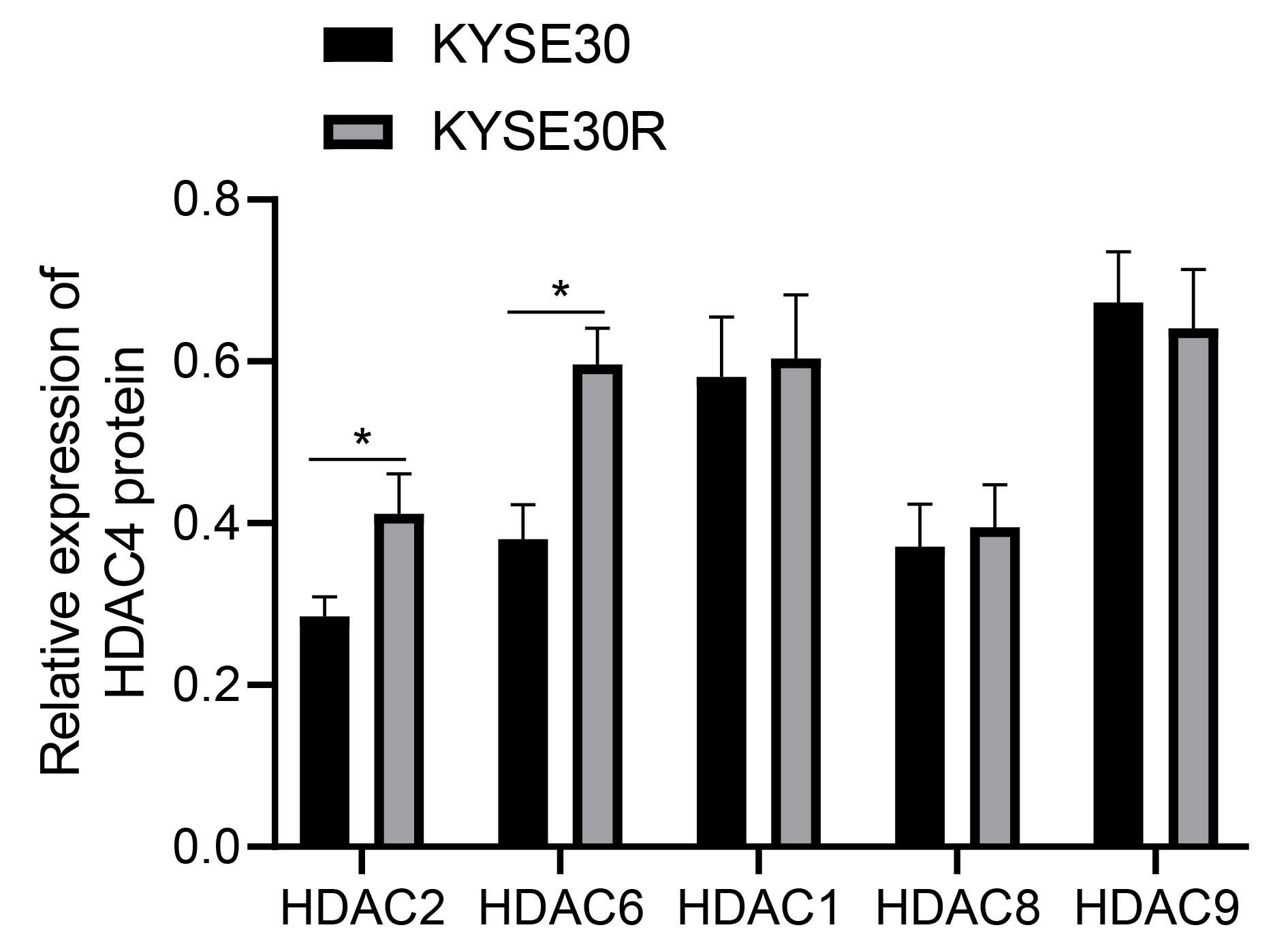

Supplement: Supplementary file 2 — Additional file 2: Figure S1 Representative Western blots for Fig. 1H (A), 2A (B), 4D (C), 4G (D), 5A (E), and 6A (F). [file 12967_2021_3171_MOESM2_ESM.jpg]

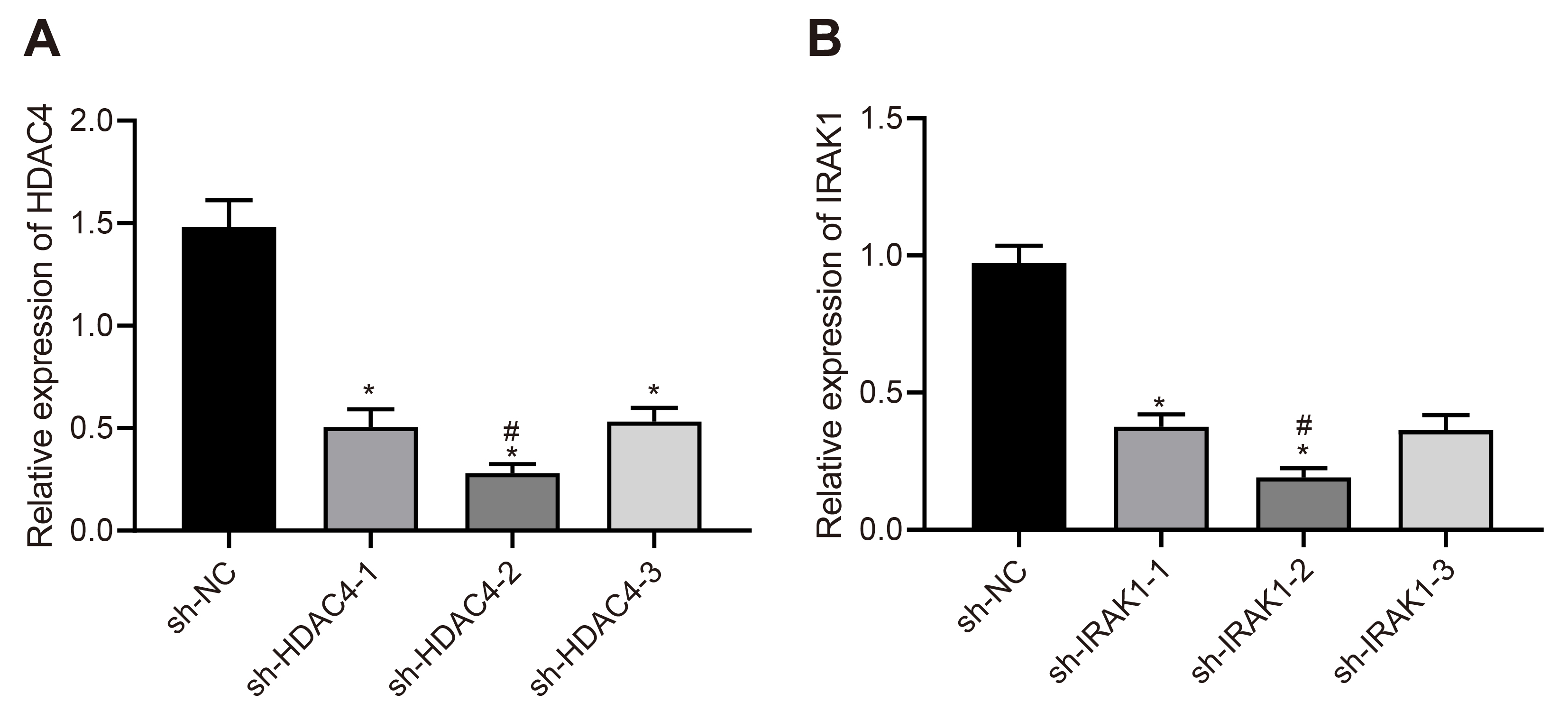

Supplement: Supplementary file 3 — Additional file 3: Figure S2 Expression of HDAC1, HDAC2, HDAC6, HDAC8 and HDAC9 in KYSE30 and radioresistant KYSE30 (KYSE30R) cell lines was determined by Western blot analysis. Values obtained from three independent experiments in triplicate were expressed as mean ± standard deviation. Values among three or more groups were compared by one-way ANOVA followed by Tukey's post hoc test. * p < 0.05 compared with KYSE30 cells. [file 12967_2021_3171_MOESM3_ESM.jpg]

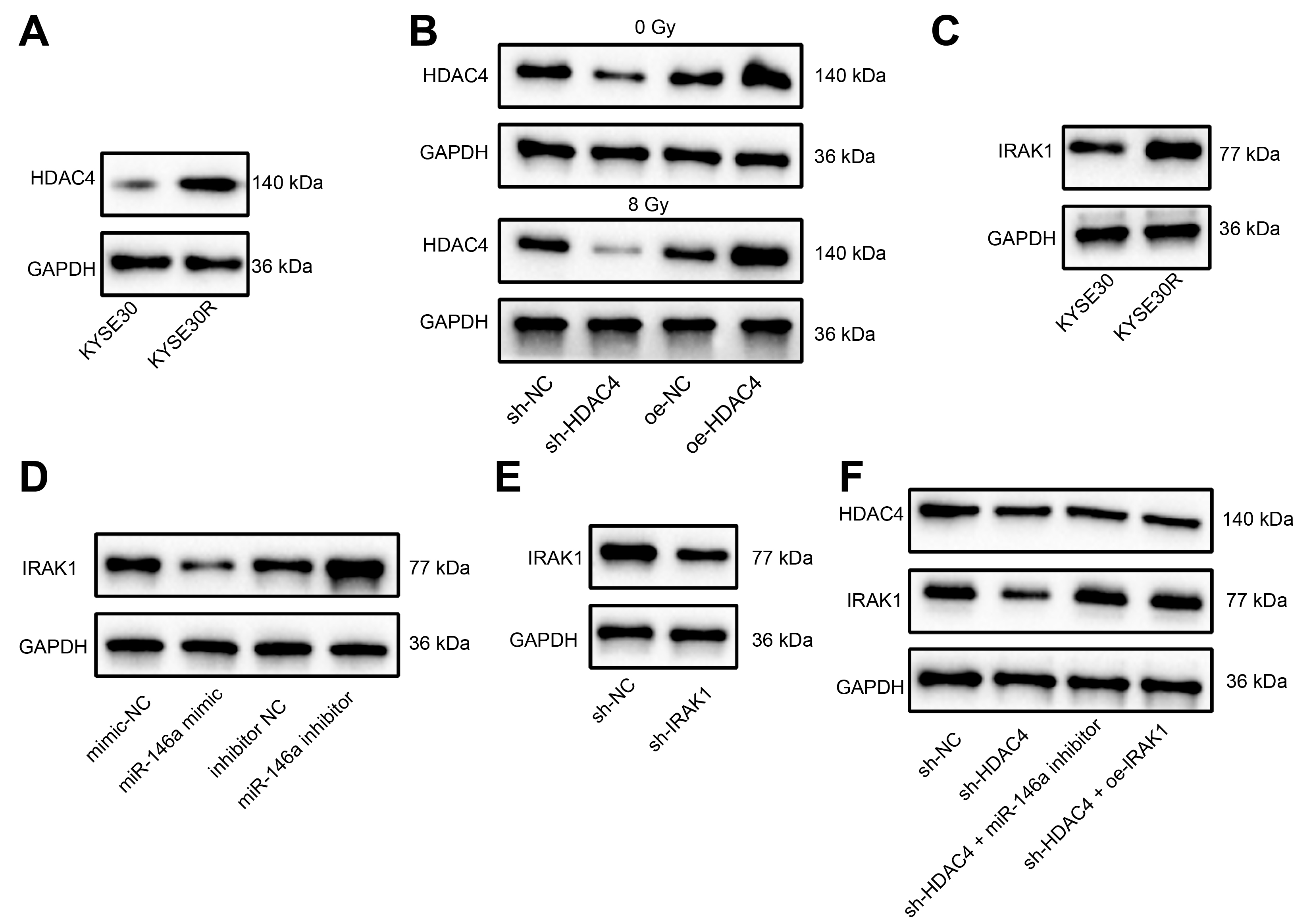

Supplement: Supplementary file 4 — Additional file 4: Figure S3 Silencing efficiency of HDAC4 and IRAK1. (A) HDAC4 knockdown was determined by Western blot analysis, revealing sh-HDAC4-2 with best silencing efficiency. (B) IRAK1 knockdown was determined by Western blot analysis, revealing sh-IRAK1-3 with best silencing efficiency. Values obtained from three independent experiments in triplicate were expressed as mean ± standard deviation. Values among three or more groups were compared by one-way ANOVA followed by Tukey's post hoc test. * p < 0.05 compared with sh-NC. # p < 0.05 compared with sh-HDAC4-1 and sh-HDAC4-3 or sh-IRAK1-1 and sh-IRAK1-2. [file 12967_2021_3171_MOESM4_ESM.jpg]

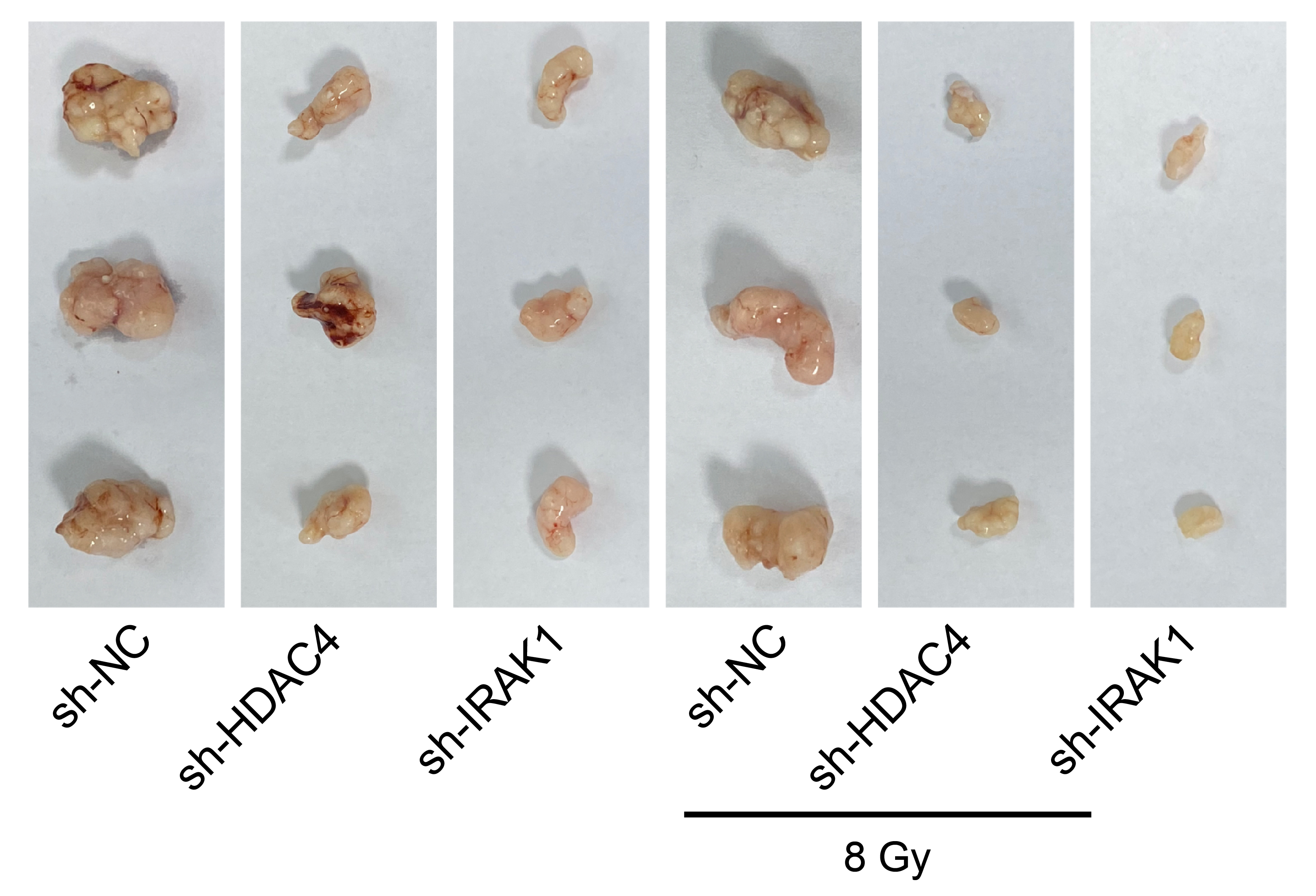

Supplement: Supplementary file 5 — Additional file 5: Figure S4 Representative images of tumors collected from nude mice xenografted with radioresistant KYSE30 cells in response to sh-HDAC4 or sh-IRAK1. These mice were unirradiated or received local X-ray irradiation at a dose of 8 Gy. [file 12967_2021_3171_MOESM5_ESM.jpg]
